# An Overview of the Molecular Methods in the Diagnosis of Gastrointestinal Infectious Diseases

**DOI:** 10.1155/2020/8135724

**Published:** 2020-03-24

**Authors:** Muhammad Amjad

**Affiliations:** Department of Clinical Laboratory Science, Marshall University, 1 John Marshall Drive, Huntington, WV 25755, USA

## Abstract

Gastrointestinal infectious diseases are very common worldwide and an important cause of morbidity and mortality, particularly in infants in developing countries. Diarrhea and other intestinal infections are caused by a wide range of bacteria, viruses, protozoa, and parasites. Conventional diagnosis of these infections is performed by culture, microscopy, and antigen detection immunoassays. The traditional culture and microscopy procedures are time-consuming, lack sensitivity, and require special laboratory setup and well-trained staff. However, based on the advancement in the molecular diagnostics and with the introduction of commercially available tests, traditional diagnostic techniques have been continuously replaced by these newer rapid antigen detection and molecular-based methods. This review summarizes and discusses the availability, advantages, and disadvantages of molecular methods in the detection and identification of human gastrointestinal pathogens.

## 1. Introduction

Gastrointestinal infections are among the most common infectious diseases worldwide, being exceeded only by respiratory tract infections [1]. Gastrointestinal infections are caused by a variety of bacterial, viral, and parasitic pathogens. These infections are mostly transmitted in poor hygienic conditions and by consuming contaminated food or water. Infections can also be transmitted from person to person by direct contact or through fomites. The most common symptom is diarrhea, which is usually a self-limiting disease, and most of the healthy individuals recover within few days. However, in very young patients, and with poor hygiene, diarrheal disease may progress, leading to severe dehydration, malnourishment, bacteremia, and other complications that may lead to death [2]. According to the report of the World Health Organization (WHO), there are over 1.7 billion cases of diarrheal disease worldwide every year [3]. Furthermore, diarrheal diseases are the second leading cause of death in children under five years of age [3].

The incidence rate and mortality from gastrointestinal infections and diarrhea are relatively low in developed countries including the United States. However, these illnesses remain a major public health burden. In the US, 211–375 million cases of diarrheal disease are estimated each year, including 1.8 million hospitalizations, and up to 6,000 deaths [4, 5]. Furthermore, in the United States, the cost of hospitalization due to gastrointestinal infections exceeds 6 billion dollars annually. Early diagnosis of enteric diseases and identification of etiological agents are helpful in patient management and in making appropriate treatment decisions. Furthermore, it is also very helpful for infection control from a public health point of view.

The important causative agents of bacterial gastroenteritis and diarrhea are *Campylobacter*, *Salmonella*, *Shigella*, *Plesiomonas*, *Vibrio*, *Yersinia enterocolitica*, *Clostridioides difficile* (formerly *Clostridium difficile*), and pathogenic strains of *Escherichia coli* [4, 6]. Important causative agents of viral gastroenteritis are Adenovirus, Rotavirus, Astrovirus, and Norovirus [4, 6]. Parasitic infections are caused by a variety of helminths, protozoa, ciliates, and coccidian organisms. Acute gastrointestinal infections with diarrhea are mostly caused by *Giardia lamblia*, *Entamoeba histolytica*, *Cyclospora cayetanensis*, and *Cryptosporidium* species [7].

Recent development in the field of Molecular Diagnostics and the availability of commercial Nucleic Acid Amplification Techniques (NAATs) based assays have changed the way we used to perform laboratory diagnosis of enteric infections. This review summarizes the currently available Food and Drug Administration- (FDA-) approved and some commonly available European In Vitro Diagnostic Devices (CE-IVD) marked molecular methods for the diagnosis of gastrointestinal infectious diseases. Advantages and disadvantages are discussed to see if we are ready to move from traditional and immunological based assays to molecular methods.

## 2. Traditional Culture, Microscopy, and Immunological Techniques

Traditional laboratory diagnoses of gastrointestinal infections and enteric pathogens detection are performed by (1) culture and antibiotic susceptibility testing, (2) ova and parasite microscopy examination, and (3) antigen detection via immunoassays.

In the clinical and diagnostic microbiology, assay sensitivity and specificity are important parameters and are used in the evaluation of a newly developed test after comparison with a reference gold standard method. A test or a newly developed test validation sensitivity is the ability of a test to correctly identify those with the disease (true positive), whereas test specificity is the ability of the test to correctly identify those without the disease (true negative). When a newly developed test is evaluated by comparison with a nonreference method, the terms sensitivity and specificity are not used. Rather numerical calculations are called as positive percent agreement (PPA) and negative percent agreement (NPA) instead of sensitivity and specificity. While sensitivity and specificity are characteristics of a test, two other parameters, positive predictive value (PPV) and negative predictive value (NPV), are used to determine clinical relevance and effectiveness of a test in determining a specific disease in a specific patient population. PPV is the probability that following a positive test result, that individual will truly have that specific disease, and NPV is the probability that following a negative test result, that individual will truly not have that specific disease.

Conventional culture remains a gold standard for the diagnosis of bacterial enteropathogens with several advantages and disadvantages. The major advantage of the culture method is its specificity. Specificity of culture is 100% if the pathogenic organism is not found in healthy subjects. However, the sensitivity of culture varies and is usually low and more difficult to determine. Another advantage of the culture method is the availability of isolate, which may be used for further testing including antibiotic susceptibility testing. When a traditional culture method is performed, the isolate can be referred to state public health laboratories for further identification, outbreak investigations, and epidemiological studies. The disadvantage of the culture method is poor sensitivity and the fact that it requires 3–5 days for pathogen detection and finalizing reports. For traditional stool culture, virus or parasite detection, patient history, and request for specific testing may be required for the proper selection of media and method. Laboratories may lack resources, trained staff, and equipment to detect some of the pathogens in the clinical specimens. Furthermore, in case of traditional bacterial culture, time and experience are required to screen all the normal flora, look for the possible pathogen and subculture, and setup further identification procedures [8]. Ova and parasite microscopic examination of stool sample is useful in the direct detection of intestinal parasites. However, microscopic examination of the direct smear or stained smear of the stool for the intestinal parasite has low sensitivity, is technically challenging, and requires highly trained and experienced personnel [9]. Ova and parasite detection can be complicated due to low organism burden and/or intermittent shedding. Antigen detection by commercially available immunoassays is popular and easy to perform procedure to detect certain intestinal parasites and viruses. However, these antigen-based assay doses generally have low sensitivity and do not detect all the pathogens involved in gastrointestinal infections.

## 3. Nucleic Acid-Based Amplification Techniques (NAATs)

In the last decade, commercially available nucleic acid-based methods have focused on the detection of either a single pathogen or multiple pathogens in a multiplex assay format. Several molecular assays are available for the detection of a single gastrointestinal pathogen. These assays are especially designed to target specific patient population and to meet medical coding and billing requirements. Furthermore, these single NAAT assays allow for particular testing that a physician may order. Common single molecular assays are used for the detection of toxigenic *C. difficile* and Norovirus infections [1, 10–12].

Current molecular techniques include (1) polymerase chain reaction (PCR) in a real-time format, (2) endpoint PCR with microfluidics and array technologies, and (3) integrated platforms in which nucleic acid extraction, amplification, and analysis are performed in a single step. Recently, isothermal amplification, in which thermal cycling is not required, has been gaining popularity. These isothermal helicase-dependent amplification techniques do not require expensive thermal cycling equipment and are more suitable for the detection of a single pathogen.

### 3.1. Molecular Tests for the Detection of Toxigenic *Clostridioides difficile*

*Clostridioides difficile* is a major causative agent of nosocomial and antibiotic-associated diarrhea and pseudomembranous colitis [13, 14]. *C. difficile* can normally colonize the gastrointestinal tract of up to 90% of healthy newborns and infants and up to 15% healthy adult population. Risk factors for *C. difficile*-associated disease are older age, hospitalization, or stay in long-term care facilities, and typically diarrhea symptoms occur after antibiotic treatment. The pathogenicity of *C. difficile* is associated with the production of a binary toxin, and a large clostridial toxin comprising of the toxin A (TcdA) and toxin B (TcdB), and non-toxin-producing strains are considered as nonpathogenic [15]. *C. difficile* toxin A, encoded by the tcdA gene, is an enterotoxin that causes diarrhea. Toxin B, encoded by the tcdB gene, causes cellular destruction leading to pseudomembranous colitis, which may progress to the complications of the development of toxic megacolon, perforation of the colon, and sepsis. The tcdC gene regulates toxin A and B production. Genes encoding for toxins A and B are present in the pathogenicity locus (PaLoc) together with three additional genes that have been implicated in regulation (tcdR and tcdC) and secretion (tcdE) [16]. In recent years, several outbreaks have been reported with increased morbidity and mortality by a hypervirulent ribotype 027 strain of *C. difficile* (NAP1 strain) [17, 18]. This strain shows high virulence due to a base pair frameshift mutation in the regulatory tcdC gene, which leads to the increased toxin production and pathogenicity [19]. Based on the fact that *C. difficile* can normally colonize, multiplex assays for the detection of enteric pathogens discussed in this article are not recommended for the diagnosis of *C. difficile*-associated disease. According to the most recent Infectious Diseases Society of America/Society for Healthcare Epidemiology of America (IDSA/SHEA), *C. difficile* testing is recommended in high-risk adults and children of ≥2 years of age with onset of ≥3 unformed stools in 24 hours, following antimicrobial treatment, healthcare-associated diarrhea, and in patients with persistent chronic diarrhea without any etiology [20].

**Figure 1:**
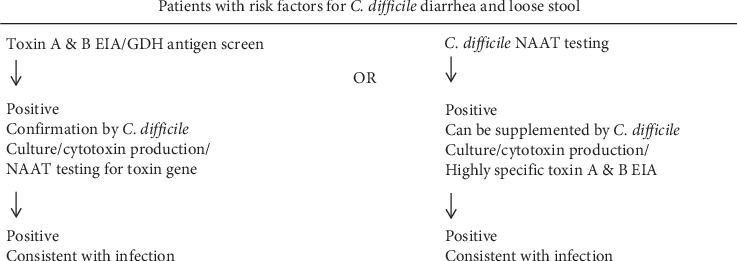
*Clostridium difficile* testing algorithm.

Laboratory diagnosis of *C. difficile* colonization and disease is performed by the demonstration of the presence of the pathogen and their toxin production in the stool samples. Initial screening of *C. difficile* colonization is performed by the antigen-based immunoassays, e.g., toxin A and B detection using chromatographic/lateral flow membrane cartridge devices or enzyme immunoassay (EIA). Initial screening can also be performed by detection of the presence of glutamate dehydrogenase (GDH) enzyme in the patient stool sample in a solid-phase microtiter plate format or in a chromatographic/lateral flow membrane cartridge device as a single test, or as a combined test with the detection of toxins A and B. GDH is a constitutive enzyme produced by all strains of *C. difficile* independent of toxigenicity, and its presence indicates colonization and not necessarily active *C. difficile*-associated disease. All positive screen results need to be further confirmed by either molecular NAAT-based assay, including PCR for the detection of the toxin-producing genes, or culture and cytotoxin production assays. Toxigenic culture involves isolation of *C. difficile* from the stool sample on cycloserine-cefoxitin-fructose agar (CCFA) and then demonstration of toxin production of the isolates by cell culture cytotoxicity assay. Cytotoxin production assay can also be used directly to detect the presence and activity of the toxin in the stool filtrates. Exposure of cell lines with cell culture supernatant or stool filtrates can typically show the cytopathic effect of the cell rounding and is due to the presence of the toxin B than toxin A. Since *C. difficile* can be normally present in stool samples, most of the institutes use a two-step reflex algorithm to determine active *C. difficile* infection (Figure 1). This two-step approach uses a combination of screen test by toxin A and B EIA/GHD antigen and confirmatory NAAT testing for the detection of toxin gene or demonstration of toxin production by toxigenic culture/cytotoxicity assay [21, 22]. The diagnosis can be performed by EIA/GDH antigen screen first and, if positive, demonstration of the presence of the toxin-producing genes by NAAT testing or toxigenic culture/cytotoxicity assay. Alternately, NAAT can be performed first, and, if positive, it usually indicates infection. However, a positive NAAT test may be because of asymptomatic colonization of toxigenic *C. difficile*, which can be further confirmed by the use of highly specific toxin A and B EIA or demonstration of toxin production by toxigenic culture/cytotoxicity assays (Figure 1).

Several FDA-approved methods are available for the diagnosis of *C. difficile* infection (Table 1). AmpliVue *C. difficile* (Quidel Diagnostics, San Diego, CA, USA) is a semiautomated assay which uses isothermal helicase-dependent amplification and hybridization in a cartridge/chip-based format. A comparative study of AmpliVue *C. difficile* with illumigene *C. difficile* and reference toxigenic culture method showed this assay sensitivity and specificity to be 96% and 100%, respectively [23]. Illumigene *C. difficile* assay (Meridian Bioscience, Cincinnati, OH, USA) is also an isothermal amplification system and uses loop-mediated isothermal amplification (LAMP) technology. In an independent study, the sensitivity and specificity of this assay were reported as 88.1% and 96.7%, respectively, as compared with the reference toxigenic culture method [24]. These integrated isothermal amplification-based assays provide an alternative to more expensive PCR-based tests and equipment and are easy to perform.

Other FDA-approved real-time PCR-based assays that detect tcdB gene include ProGastro Cd Test (Hologic, San Diego, CA, USA) using SmartCycler (Cepheid, Sunnyvale, CA, USA), Lyra *C. difficile* (Quidel, San Diego, CA, USA) using SmartCycler/ABI 7500/QuantStudio, and Simplexa *C. difficile* based on 3M Integrated Cycler (Focus Dx, Cypress, CA, USA) (Table 1). These assays require separate nucleic acid extraction step, and amplification and detection are performed on respective real-time PCR analyzers. Other assays that detect tcdB gene using integrated dedicated real-time PCR systems include BD Max *C. difficile* (Becton Dickinson, Sparks, MD, USA), Cobas Liat Cdiff (Roche Diagnostics, Indianapolis, IN, USA), and GenePOC CDiff assay (Meridian Bioscience, Cincinnati, OH, USA). These assays are based on integrated systems and do not require the separate nucleic acid extraction step. Several comparative studies with culture and other molecular methods showed these assays to have good correlation and sensitivities and specificities [25–28]. The remaining three assays, ARIES *C. difficile* (Luminex, Austin, TX, USA), GeneXpert *C. difficile*/Epi (Cepheid, Sunnyvale, CA, USA), and Verigene *C. difficile* (Luminex, Austin, TX, USA), use real-time PCR-based ARIES system, GeneXpert multiplex PCR system, and nanoparticle array hybridization-based Verigene equipment, respectively (Table 1). All of these assays use closed cartridge-based system in which nucleic acid extraction, amplification, and detection are performed simultaneously without separate processing, thus minimizing the chances of contamination and false-positive results. Several independent studies have been performed on the evaluation of these assays and have sensitivities and specificities in the range of upper 90s [25, 26, 28]. All of these assays target *C. difficile* tcdB gene, while GeneXpert *C. difficile* and Verigene *C. difficile* assays can also detect hypervirulent ribotype 027 (BI/NAP1/027) strain of *C. difficile* by targeting the regulatory tcdC gene in which there is a deletion of nucleotide at position number 117 (Δ117tcdC) [25, 26, 28, 29]. Besides, there are several CE-IVD and in-house assays available for the detection of *C. difficile* genes (Table 1). Performance characteristics of these assays are mostly performed by manufacturers, and there are limited data available from the independent studies.

### 3.2. Commercially Available Multiplex Assays for the Detection of Enteric Pathogens

There are several multiplex commercial assays available that can detect most of the common pathogens in an open system, in which separate nucleic acid extraction step is required, or closed assays and systems, in which simultaneous nucleic acid extraction, amplification, and product analysis are performed. These assays can detect pathogens that may or may not be prevalent in a setting, and local epidemiology as well as institutional need should be considered before acquisition. Some assays offer separate bacterial, viral, and parasite panels, making them flexible in situations where specific testing may have been requested by a physician. Furthermore, these separate bacterial, viral, and parasite panels can be used to resolve patient billing issues. This review article discusses current FDA-approved (Table 2) and commonly available CE-IVD marked approved (Table 3) multiplex NAAT commercial assays for the identification of enteric pathogens. For the identification of pathogens, these assays utilize multiplex PCR followed by either hybridization to microarray, hybridization probes, or melting curve analysis.

#### 3.2.1. BioFire Gastrointestinal (GI) Panel

The BioFire Gastrointestinal (GI) Panel (BioFire, Salt Lake City, UT, USA) is a fully integrated system that allows for the simultaneous detection of a greater number of bacteria (13 pathogens), viruses (5 pathogens), and parasites (4 pathogens) than other assays (Table 2). This system simultaneously performs nucleic acid extraction, reverse transcription, amplification, and analysis within one hour. The technology is based on multiplex PCR amplification followed by endpoint melting curve data analysis. The main advantage of BioFire FilmArray is its comprehensive coverage of most the pathogen and low hands-on and turnaround time. The disadvantage of filmarray is low throughput and inability to separate bacterial, viral, or parasitic testing if needed from patients need, or billing point of view. A multicenter evaluation of BioFire GI Panel with conventional stool culture and molecular methods showed the FilmArray GI Panel sensitivity to be 100% for 12 of the 22 and >94.5% for an additional 7 of the 22 target pathogens tested. For the remaining 3 targets, sensitivity could not be calculated due to the low prevalence of the pathogens in the study [30].

#### 3.2.2. Luminex Gastrointestinal Pathogen Panel (xTAG GPP)

The Luminex xTAG GPP (Luminex, Austin, TX, USA) is an FDA-approved assay which allows for the detection of 14 broad range of pathogens in a single test (9 bacterial, 3 viral, and 3 parasitic) (Table 2). Luminex xTAG GPP is not an integrated system and requires a separate nucleic acid extraction step, followed by multiplex PCR and reverse transcriptase PCR, hybridization to bead array, and detection by Luminex equipment. Luminex xTAG GPP test sensitivity is in between 90 and 100%, depending on pathogen present, and specificity in the range of 91 to 99% [31–33]. The main advantage of Luminex xTAG GPP is its high sample throughput and the ability to detect multiple pathogens. However, the major disadvantage is that it is not an integrated platform and requires separate nucleic acid extraction and post-PCR handling, which increases the potential of cross-contamination and false-positive results [31, 34].

#### 3.2.3. Verigene Enteric Pathogen (EP) Test

The Verigene Enteric Pathogen (EP) (Luminex, Austin, TX, USA) is an integrated FDA-approved system for the simultaneous detection of common stool pathogens (Table 2). This system detects up to 5 bacterial (*Campylobacter*, *Salmonella*, *Shigella*, *Vibrio*, and *Yersenia enterocolitica*), Shiga Toxin 1 (stx1), Shiga Toxin 2 (stx2), and 2 viral pathogens (Norovirus and Rotavirus) and does not cover any of the parasitic pathogens. The Verigene platform uses a processor and reader which can simultaneously perform nucleic acid extraction, amplification, and hybridization to probes on a glass slide in a microarray format. The manufacturer reported sensitivities and specificities of the test are in the range of >91% and >99%, respectively, for the target organisms. A comparative study of Verigene EP test with BioFire FilmArray GI panel and Luminex xTAG GI panel showed this assay to be less sensitive and specific as compared to BioFire Array GI panel [35].

#### 3.2.4. ProGastro SSCS Assay

The Prodesse ProGastro SSCS (Hologic, San Diego, CA, USA) is another commercially available FDA-approved assay that is used for the simultaneous detection of 4 bacterial pathogens (*Campylobacter*, *Salmonella*, *Shigella*, and Shiga toxin-producing *E. coli* (STEC) stx1 and stx2 genes) (Table 2). ProGastro SSCS is not an integrated system and requires a separate nucleic acid extraction step, followed by PCR amplification in SmartCycler (Cepheid, Sunnyvale, CA, USA) and data analysis. The overall sensitivity of this assay is 98.5% and specificity is in the range of 98.9% to 99.4%, depending on the target pathogen [1, 36].

#### 3.2.5. BD Max Enteric and Extended Enteric Panels

The BD Max (Becton Dickinson, Sparks, MD, USA) is an integrated system that incorporates simultaneous sample preparation, nucleic acid extraction, amplification, and detection. BD Max microfluidic real-time PCR-based system batches up to 24 samples within 3 hours and required 2 minutes of hands-on time per sample. BD Max Enteric panel is an FDA-approved assay that can be used to detect 5 bacterial pathogens, i.e., *Campylobacter*, *Salmonella*, *Shigella*, Enteroinvasive *E. coli* (EIEC), and Shiga toxin-producing *E. coli* (STEC) stx1 and stx2 genes (Table 2). A comparison of BD Max enteric panel testing with the conventional culture method showed increased sensitivity and specificity in the detection of *Campylobacter*, *Salmonella*, *Shigella*, and STEC [37, 38]. The other BD Max panels include the extended enteric bacterial panel that can detect (*Yersinia enterocolitica*, *V. cholera*, *V. parahaemolyticus*, and *V. vulnificus*), viral panel (Adenovirus, Astrovirus, Norovirus, and Sapovirus), and parasite panel (*C. parvum*, *C. hominis*, *Entamoeba histolytica*, and *Giardia lamblia*) [39].

#### 3.2.6. Allplex Gastrointestinal Panel

The Allplex Gastrointestinal Assays (Seegene, Seoul, South Korea) is a new CE-IVD marked multiplex real-time PCR assay that detects 13 bacteria, 5 viruses, and 6 parasites in 4 multiplex PCRs (Table 3). This assay uses the novel analytical multiple detection temperature (MuDT) technique which is able to detect multiple targets in a single fluorescence channel without melting curve analysis. The procedure involves separate nucleic acid extraction from stool samples, followed by multiplex real-time PCR using the CFX96TM real-time PCR system (Bio-Rad Laboratories, Richmond, CA, USA) and detection by data analysis. Two comparative studies with routine methods showed Allplex Gastrointestinal multiplex PCR assay to be more sensitive and specific as compared to traditional methods [40, 41]. A comparative evaluation and laboratory performance of Seegene Allplex Gastrointestinal with the conventional procedure and two other NAT methods showed this assay to have an overall 94% positive percent agreement for the detection of gastrointestinal pathogens [42].

#### 3.2.7. Seeplex Diarrhea ACE Detection

The Seeplex Diarrhea ACE Detection kits (Seegene, Seoul, Korea) for Bacteria 1, Bacterial 2, and Virus are CE-IVD-approved panels, and these multiplex PCR-based kits allow the detection of common bacterial and viral pathogens [43]. This multiplex PCR assay enables simultaneous multipathogen detection of 9 bacteria, 4 viruses, and a *C. difficile* toxin-producing gene using three multiplex assays (Table 3). The test procedure includes separate nucleic acid extraction, reverse transcription followed by PCR, and product separation by capillary electrophoresis. The major disadvantages of these kits are that separate nucleic acid extraction is required and none of the parasitic pathogens can be detected. The sensitivity of these assays is in the range of 40–100%, and specificity is in the range of 96–100% depending on the pathogen present in the sample [43–45].

#### 3.2.8. RIDA GENE Real-Time PCR Kits

The RIDA GENE gastrointestinal kits (R-Biopharm AG, Darmstadt, Germany) offers real-time PCR-based separate bacterial, viral, and parasitic panel that can detect a range of common pathogens (Table 3). These CE-IVD diagnostic tests require separate nucleic acid isolation procedure and can be performed on most commonly available real-time PCR equipment. A comparative study of RIDA GENE Bacterial Stool and two other molecular methods, the Fast Track Diagnostics (FTD) Bacterial Gastroenteritis Panel and the BD MAX Enteric Bacterial Panel, indicates RIDA GENE gastrointestinal to be more sensitive than culture methods for the detection of *Campylobacter* and *Shigella* species [46]. However, the sensitivity of RIDA GENE GI Kit for the detection of *Salmonella* spp. was found to be low at 25% as compared to the culture method [1, 46].

#### 3.2.9. FTD Bacterial Gastroenteritis Panel

The Fast Track Bacterial GI panel (Fast Track Diagnostics, Junglinster, Luxembourg) is a CE-IVD marked two-tube multiplex real-time PCR test for the detection of pathogen genes by TaqMan technology using the ABI 7500 Fast instrument (Applied Biosystems, Foster City, CA, USA). This system requires separate nucleic acid extraction step. The first tube performs multiplex detection of *Campylobacter coli/jejuni/lari* and Enterohemorrhagic *E. coli*. Second tube performs multiplex PCR detection of *Salmonella* spp., *Shigella*/Enteroinvasive *E. coli*, *Yersinia enterocolitica*, and *C. difficile*. Validation and performance characteristics of these assays are determined by the manufacturer. One comparative study of FTD Bacterial GI panel, RIDA GENE GI, and BD Max showed FTD GI panel to be more sensitive than culture methods for the detection of *Campylobacter* and *Shigella* species. However, for *Salmonella* spp., FTD Bacterial GI panel showed a low sensitivity of 50% as compared to the culture method [46].

#### 3.2.10. EntericBio Gastro Panels

The EntericBio Gastro panels (Serosep, Limerick, Ireland) are CE-IVD assays that offer several bacterial, viral, parasite, and combo panels that cover most of the enteropathogens (Table 3). Compared with the previous version and culture methods, the sensitivity and specificity of these assays are reported to be in the range of 100% and 97.8%, respectively [47, 48]. These assays require separate nucleic acid extraction, followed by real-time PCR amplification by LightCycler 480 II (Roche Diagnostics, Indianapolis, IN, USA) instrument and data analysis.

#### 3.2.11. QIAstat-Dx Gastrointestinal Panel

The QIAstat GIP (Qiagen, Hilden, Germany) is a new multiplex PCR assay that can simultaneously detect and identify 24 gastroenteritis pathogens from stool samples in Cary-Blair transport medium (Table 3). QIAstat GIP is an integrated system and uses cartridge and QIAstat-Dx analyzer, in which nucleic acid extraction, real-time PCR amplification, and fluorescent amplicon detection are performed in a closed system. Manufacturer reports the overall assay sensitivity and specificity to be 97.9% and 97.8%, respectively. A multicenter comparative study of QIAstat GIP with BioFire FilmArray GIP and Seegene Allplex GIP indicates a good correlation and positive percent agreement of 98.2% with these other assays [49].

#### 3.2.12. CLART EnteroBac

The CLART EnteroBac (Genomica, Madrid, Spain) is a PCR array-based system that simultaneously allows detection and identification of 8 bacterial pathogens (Table 3). The test procedure includes nucleic acid extraction, multiplex PCR amplification, microarray hybridization, and automated data analysis. The advantage of this assay is high throughput and disadvantage is that it does not detect any viral and parasitic pathogens. Validation and performance characteristics of this assay are performed by manufacturer, and there are limited independent studies available.

#### 3.2.13. GastroFinder 2SMART

The GastroFinder 2SMART assay (PathoFinder, the Netherlands) is a CE-IVD real-time PCR-based assay which is able to detect 9 bacterial, 5 viral, and 4 parasitic pathogens causing gastrointestinal infection in one multiplex assay (Table 3). This is not an integrated assay and requires separate nucleic acid extraction followed by real-time PCR amplification and identification of organisms on the basis of melting curve analysis. Performance of this assay is evaluated by the manufacturer with limited independent studies.

The other non-FDA and non-CE-IVD assays are EasyScreen Enteric assay (Genetic Signature's, Sydney, Australia) and Faecal Pathogens M detection assay (AusDiagnostics, Mascot, Australia). The Genetic Signature EasyScreen Enteric assay uses company's 3base technology to convert all cytosine bases (C) in the starting nucleic acid samples to thymidine (T). The resulting reduction in sequence variation allows for a higher number of multiplex targets to be run under similar conditions. Separate panels are available to detect common bacterial, viral, parasitic pathogens, and *C. difficile* including hypervirulent 027 and 078 strains. Several studies have been performed on the detection and identification of *Blastocystis* spp., *Cryptosporidium* spp., *Dientamoeba fragilis*, *Entamoeba* spp., and *Giardia lamblia* in human clinical samples [50–52]. The EasyScreen Enteric Parasite Detection Kit exhibited 92–100% sensitivity and 100% specificity [53]. AusDiagnostics Faecal Pathogens M detection assay can detect 14 common bacterial, viral, and parasitic pathogens. Faecal Pathogens M detection assay uses multiplexed tandem PCR (MT-PCR) technique comprising of two amplification steps. In the first step, extracted nucleic acid is preamplified as a single well multiplex reaction. The amplified product in the first step is diluted, and second step multiplex real-time PCR is performed using SYBR green dye, and identification of organisms is performed by melting curve analysis.

#### 3.2.14. Advantages of Molecular Testing

The main advantages of molecular testing are improved workflow and faster turnaround time with high sensitivity and specificity as compared to traditional methods. An additional advantage is the capability of multiplexing, which allows for the simultaneous detection of multiple enteric pathogens.

Multiplex assays can be particularly helpful for severely ill patients and in certain patient population where rapid diagnosis, treatment, and management decision are required. Multiplex molecular assays are helpful from the therapeutic point of view to avoid inappropriate and unnecessary antimicrobial treatment, for example, in case of Shiga toxin-producing *E. coli* (STEC) infection where antimicrobial exposure may increase the risk of patient developing hemolytic uremic syndrome (HUS). Multiplex NAAT assays can detect a variety of enteric pathogens, thus eliminating the need to stock special media and perform separate parasitology and virology testing. From public health and infection control point of view, rapid detection can be helpful in the infection control measure and prevention and spread of infections.

Because of the increased sensitivity and specificity and multiplex detection, several studies have reported increased rate in the detection of enteric pathogens as compared to traditional methods. Furthermore, there are reports of increased detection of multiple pathogens (more than one) from single specimens and in identifying coinfections [30, 49]. This increased detection of multiple pathogens may be beneficial and indicates coinfection. Multiplex gastrointestinal pathogen detection has been particularly found to be useful in one infection control study in which 22.2% of patients with negative conventional tests for *C. difficile* and/or rotavirus had the unsuspected gastrointestinal pathogen detected leading to more rational patient isolation and prevention of the nosocomial transmission [54].

#### 3.2.15. Disadvantages of Molecular Testing

**Figure 2:**
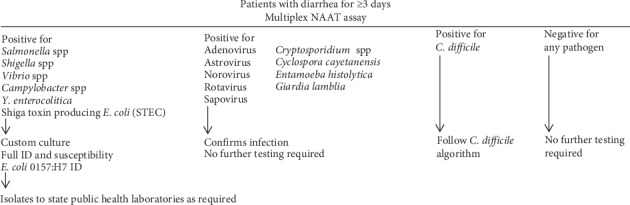
Multiplex NAAT algorithm for enteric pathogens.

The main disadvantage of NAAT is the initial setup and cost, but in the longer run replacing conventional culture methods with molecular methods is mostly beneficial. Another disadvantage is that NAAT cannot differentiate between dead and living organisms and results need to be interpreted carefully depending on the patient condition [55]. Furthermore, depending on the patient, the physician's need, and public health department requirements, a custom culture may be necessary to identify the pathogen and perform antibiotic susceptibility. The availability of antibiotic susceptibility profile is very helpful especially in critical conditions to determine if antibiotic treatment is necessary and which antibiotic to be used. Submission of selected bacterial isolates to state public health laboratories is a requirement and plays an important in the public health surveillance, outbreak investigations, and monitoring the antibiotic susceptibilities. In order to fulfil regulations, it may be necessary for labs to communicate with state labs to get approved protocol for reporting in case of NAAT testing on stool samples. Based on these complexities and individual hospital/laboratory needs, a custom multiplex NAAT algorithm can be used to determine if further testing by conventional culture method is required (Figure 2). A custom culture for identification and susceptibility testing may be required for bacterial isolates, and further identification of pathogenic *E. coli* can be performed [56]. In general, multiplex NAAT procedure should not be used for *C. difficile*. However, if the patient is found to be positive for *C. difficile* using multiplex NAAT method, separate *C. difficile* algorithm should be followed. No further testing is required if a patient is positive for viral and parasitic pathogens by NAAT test, as usually it confirms infection.

In conclusion, NAAT-based technologies provide better options in the diagnosis of infectious gastroenteritis caused by a wide range of pathogens and overcoming some of the challenges faced in the traditional microbiological and culture methods. High throughput, sensitivity, and specificity of molecular-based testing allow rapid diagnosis, treatment, and management of gastrointestinal infections. With the improvement in the technology and availability of commercially available methods, traditional laboratory diagnostic techniques for the diagnosis of gastrointestinal infectious diseases have rapidly been replaced by these newer molecular methods.

## Figures and Tables

**Table 1:** Molecular tests for detection of toxigenic *Clostridioides difficile*.

Assay		Manufacturer	Devise/analyzer	Targets/methodology
AmpliVue^®^*C. difficile*	FDA/CE	Quidel Diagnostics, San Diego, CA, USA	Hand-held Disposable cassette	tcdA, isothermal helicase-dependent
Illumigene^™^ (Alethia) *C. difficile*	FDA/CE	Meridian Bioscience, Cincinnati, OH, USA	Incubator/reader	tcdA, loop-mediated isothermal amplification (LAMP)
ProGastro^™^ Cd test	FDA/CE	Hologic, San Diego, CA, USA	SmartCycler	tcdB, real-time PCR
Lyra^®^*C. difficile*	FDA/CE	Quidel, San Diego, CA, USA	SmartCycler, ABI 7500, QuantStudio	tcdA and tcdB, real-time PCR
Simplexa^™^*C. difficile*	FDA/CE	Focus Dx, Cypress, CA, USA	3M Integrated Cycler	tcdB, real-time PCR
BD Max^™^*C. difficile*	FDA/CE	Becton Dickinson & Co., Sparks, MD, USA	BD Max System	tcdB, real-time PCR
Cobas^®^ Liat Cdiff	FDA/CE	Roche Diagnostics, Indianapolis, IN, USA	Cobas Liat System	tcdB, real-time PCR
GenePOC^™^ CDiff assay	FDA/CE	Meridian Bioscience, Cincinnati, OH, USA	Revogene integrated analyzer	tcdB, real-time PCR
ARIES^®^*C. difficile*	FDA/CE	Luminex, Austin, TX, USA	ARIES system	tcdA, tcdB, real-time PCR
GeneXpert^®^*C. difficile*/Epi	FDA/CE	Cepheid, Sunnyvale, CA, USA	GeneXpert system	tcdB, Δ117tcdC (BI/NAP1/027, real-time PCR
Verigene^®^*C. difficile*	FDA/CE	Luminex, Austin, TX, USA	Verigene processor and reader	tcdA, tcdB, Δ117tcdC (BI/NAP1/027), multiplex PCR/nanoparticle array hybridization
Qiagen^®^ artus *C. difficile* QS-RGQ	FDA/CE	Qiagen, Hilden, Germany	QIAsymphony rotor-gene Q instruments	tcdA, tcdB, real-time PCR
EntericBio real-time^®^*C. difficile*	CE-IVD	Serosep, Limerick, Ireland	ABI 7500, LightCycler 480	tcdB, real-time PCR

**Table 2:** Comparative summary of commercial enteropathogen multiplex PCR assays.

Product name	BioFire® FilmArray® Gastrointestinal (GI) Panel FDA/CE-IVD	xTAG® Gastrointestinal Pathogen Panel FDA/CE-IVD	Verigene® Enteric Pathogens TestFDA/CE-IVD	Prodesse® ProGastro™ SSCS assay FDA/CE-IVD	BD MAX™ Enteric Bacterial, Ext Bacterial, Parasite, and Viral Panels FDA/CE-IVD	Stool Bacterial Pathogens Panel FDA/CE-IVD
Manufacturer	BioFire, Salt Lake City, UT, USA	Luminex, Austin, TX, USA	Luminex, Austin, TX, USA	Hologic, San Diego, CA, USA	BD, Sparks, MD, USA	Great Basin Scientific, Salt Lake City, UT, USA
*Bacteria*						
*Campylobacter* spp	√*C. jejuni, C. coli, C. upsaliensis*	√	√*C. jejuni, C. coli, and C. lari*	√*C. jejuni, C. coli*	√	√*C. jejuni, C. coli*
*Clostridioides difficile*	√Toxin A/B	√Toxin A/B				
*Plesiomonas shigelloides*	√					
*Salmonella* spp	√	√	√	√	√	√
*Yersinia enterocolitica*	√	√^*∗*^	√		√	
*Vibrio* spp	√*V. cholera*, *V. parahaemolyticus, V. vulnificus*	√*V. cholera*	√*V. cholera, V. parahaemolyticus*		√*V. cholera, V. parahaemolyticus, V. vulnificu*s	
*Enteroaggregative E. coli* (EAEC)	√					
*Enteropathogenic E. coli* (EPEC)	√					
*Enterotoxigenic E. coli* (ETEC) *lt/st*	√	√			√	
*Shiga-like toxin-producing E. coli* (STEC) *stx1/stx2*	√		√	√	√	√
*E. coli* O157	√	√				√
*Enteroinvasive E. coli* (EIEC)/*Shigella* spp	√	√	√*S. dysenteriae, S. boydii, S. sonnei, and S. flexneri*	√	√	√*Shigella* spp
*Viruses*						
Adenovirus	√F40/41	√F40/41			√F40/41	
Astrovirus	√				√	
Norovirus	√GI/GII	√GI/GII	√GI/GII		√	
Rotavirus	√A	√A	√A		√A	
Sapovirus	√I, II, IV, and V				√	
*Parasites*					√F40/41	
*Cryptosporidium* spp	√	√			√*C. parvum, C. hominis*	
*Cyclospora cayetanensis*	√					
*Entamoeba histolytica*	√	√			√	
*Giardia lamblia*	√	√			√	

^*∗*^Not available in the United States.

**Table 3:** Comparative summary of commercial enteropathogen multiplex PCR testing kit.

Product name	Allplex^™^ Gastrointestinal Panel (virus, bacteria 1, bacterial 2, and parasite) CE-IVD	Seeplex^®^ Diarrhea ACE Detection (virus, bacteria 1, and bacterial 2) CE-IVD	QIAstat-Dx^®^ Gastrointestinal Panel CE-IVD	RIDA^®^ GENE real-time PCR kits (bacterial, parasite, and viral stool) panel CE-IVD	EntericBio real-time^®^ Gastro Panel I, II and III. Virus panel CE-IVD	CLART^®^ EnteroBac CE-IVD	GastroFinder^®^ 2SMART CE-IVD
Manufacturer	Seegene, Seoul, South Korea	Seegene, Seoul, South Korea	Qiagen, Hilden, Germany	R-Biopharm AG, Darmstadt, Germany	Serosep, Limerick, Ireland	Genomica, Madrid, Spain	PathoFinder, the Netherlands
*Bacteria*							
*Aeromonas spp.*	√	√*A. media, A. veronii*, *A. salmonicida*, *A. sobria, A. bivalvium*, *A. hydrophila*				√	
*Campylobacter* spp.	√	√*C. jejuni*, *C. coli*	√*C. jejuni*, *C. coli*, *C. upsaliensis*	√	√	√*C. jejuni, C. coli*, *C. lari*, *C. laridis*, *C. upsaliensis*	√
*Clostridium difficile*	√Toxin B and hypervirulent stain	√Toxin B	√Toxin A/B		√	√Toxin B	√Toxins A and B
*Clostridium perfringens*		√					
*Plesiomonas shigelloides*			√				
*Salmonella spp.*	√	*√S. bongori*, *S. enterica*	√	√	√	√	√
*Yersinia enterocolitica*	√	√	√		√	√*Y. enterocolitica*, *Y*. *pestis*, *Y. pseudotuberculosis*	√
*Vibrio cholerae and other Vibrio* spp.	√	√*V. cholerae*, *V. parahaemolyticus*, *V. vulnificus*	√*V. cholerae*, *V parahaemolyticus, V. vulnificus*		√		
*Enteroaggregative E. coli* (EAEC)	√		√				
*Enteropathogenic E. coli* (EPEC)	√		√			√	√
*Enterotoxigenic E. coli* (ETEC) *lt/st*	√		√			√	√
*Shiga-*like toxin-producing *E. coli* (STEC) *stx1/stx2*	√	√	√		√		√
*E. coli* O157	√	√O157 : H7	√		√	√	
*Enteroinvasive E. coli* (EIEC)/*Shigella* spp.	√	√*S. flexneri*, *S. boydii*, *S. sonnei*, *S. dysenteriae*	√	√	√	√*S. flexneri*, *S. boydii*, *S. sonnei*, *S. dysenteriae*	√
*Viruses*							
Adenovirus	√	√	√F40/41	√	√		√F40/41
Astrovirus	√	√	√		√		√
Norovirus	√GI/GII	√GII	√GI/GII	√	√GI/GII		√GI/GII/IV
Rotavirus	√A	√A	√A	√	√A		√A
Sapovirus	√		√ (I, II, IV, V)		√		√ (I, II, IV, V)
*Parasites*							
*Blastocystis hominis*	√						
*Cryptosporidium* spp.	√		√	√	√		√
*Cyclospora cayetanensis*	√		√				
*Dientamoeba fragilis*	√			√			√
*Entamoeba histolytica*	√		√	√	√		√
*Giardia lamblia*	√		√	√	√		√
